# Characteristics and possible mechanisms of 46, XY differences in sex development caused by novel compound variants in *NR5A1* and *MAP3K1*

**DOI:** 10.1186/s13023-021-01908-z

**Published:** 2021-06-10

**Authors:** Yiping Cheng, Jing Chen, Xinli Zhou, Jiangfei Yang, Yiming Ji, Chao Xu

**Affiliations:** 1 Department of Endocrinology and Metabolism, Shandong Provincial Hospital, Cheeloo College of Medicine, Shandong University, Jinan, 250021 Shandong China; 2 Department of Child Health, Women and Children’s Hospital, School of Medicine, Xiamen University, Xiamen, Fujian China; 3 Department of Endocrinology and Metabolism, Shandong Provincial Hospital Affiliated to Shandong First Medical University, Jinan, Shandong China; 4 Department of Radiology, Shandong Provincial Hospital, Cheeloo College of Medicine, Shandong University, Jinan, 250021 Shandong China; 5 Institute of Endocrinology, Shandong Academy of Clinical Medicine, Jinan, 250021 Shandong China; 6 Shandong Clinical Medical Center of Endocrinology and Metabolism, 324, Jing 5 Road, Jinan, 250021 Shandong China; 7 Department of Child Health, Xiamen Maternal and Child Health Hospital, Xiamen, Fujian China

**Keywords:** 46, XY differences in sex development, *NR5A1*, *MAP3K1*, Diagnosis, Heterogeneity

## Abstract

**Background:**

Dozens of genes are involved in 46, XY differences in sex development (DSD). Notably, about 3/4 of patients cannot make a clear etiology diagnosis and single gene variant identified cannot fully explain the clinical heterogeneity of 46, XY DSD.

**Materials and methods:**

We conducted a systematic clinical analysis of a 46, XY DSD patient, and applied whole-exome sequencing for the genetic analysis of this pedigree. The identified variants were analyzed by bioinformatic analysis and in vitro studies were performed in human embryonic kidney 293T (HEK-293T) cells which were transiently transfected with wild type or variant *NR5A1* and *MAP3K1* plasmid. Furthermore, protein production of SRY-box transcription factor 9 (SOX9) was analyzed in cell lysates.

**Results:**

A novel *NR5A1* variant (c.929A > C, p. His310Pro) and a rare *MAP3K1* variant (c.2282T > C, p. Ile761Thr) were identified in the proband, whereas the proband's mother and sister who only carry rare *MAP3K1* variant have remained phenotypically healthy to the present. These two variants were predicted to be pathogenic by bioinformatic analysis. In vitro, *NR5A1* variant decreased the SOX9 production by 82.11% compared to wild type *NR5A1*, while *MAP3K1* variant had little effect on the SOX9 production compared to wild type *MAP3K1*. Compared to wild type *NR5A1* transfection, the SOX9 production of cells transfected with both wild type plasmids decreased by about 17.40%. Compared to variant *NR5A1* transfection, the SOX9 production of cells transfected with both variant plasmids increased by the 36.64%.

**Conclusions:**

Our findings suggested the novel compound variants of *NR5A1* and *MAP3K1* can alter the expression of SOX9 and ultimately lead to abnormality of sex development.

**Supplementary Information:**

The online version contains supplementary material available at 10.1186/s13023-021-01908-z.

## Background

46, XY differences in sex development (46, XY DSD; ORPHA 251510) refers to the abnormal sexual differentiation process of 46, XY males caused by different reasons, and the phenotypic sex and/or appearance of gonads are inconsistent with chromosomal sex [1]. 46, XY DSD is a significant cause of birth defects, which not only brings physical and mental harm to patients, but also burdens the family. Data on the incidence of 46, XY DSD are rare and estimates vary widely. Androgen insensitivity syndrome (AIS) is thought to be the most common etiology of 46, XY DSD followed by gonadal dysgenesis. The incidences of AIS and gonadal dysgenesis are reported to be 1–5 per 100 000 births and 1 per 80 000 births, respectively [2]. 46, XY DSD is a group of diseases with highly heterogeneous phenotype: the abnormal external genitalia can be completely male or completely female, and more often, it is manifested as a phenotype between female and male, such as clitoral enlargement, hypospadias, labia majora fusion, and undescended testicles [3]. Therefore, clinical diagnosis is very difficult and more than 50% of cases could not be accurately diagnosed [4]. Genetic analysis can quickly and accurately detect the etiology of DSD, but with high cost and low penetration rate about 3/4 of 46, XY DSD cannot make an unambiguous etiology diagnosis [5]. NGS panels or whole-exome sequencing (WES) can explain 30% of case, that is to say, a lot are unexplained for clinicians. In addition, the genetic background of 46, XY DSD is complex, involving dozens of genes as *AR*, *NR5A1*, *MAP3K1*, *SRD5A2*, etc. [6], and single variant identified cannot fully explain the phenotype heterogeneity of 46, XY DSD.

The *NR5A1* gene (OMIM 184,757) plays a key role in human sex differentiation, sexual development and steroidogenesis [7]. It encodes steroidogenic factor 1 (SF-1) that controls multiple steps of adrenal gland and gonadal development: SF-1 in fetal testicular support cells actively regulates the expression of two genes, *SRY-box transcription factor 9* (*SOX9*) and *Anti-Müllerian hormone* (*AMH*), which are involved in sex determination and differentiation in male [8, 9]; SF-1 in Leydig cells controls the expression of various enzymes required for steroidogenesis, such as STAR, CYP11A1 and CYP17A1, which ultimately leads to gonad masculinization [10, 11]. The pathogenic *NR5A1* variations are thought to account for 9–10% of 46, XY DSD [12–14] and are closely related to the diverse phenotype of 46, XY DSD. Patients with *NR5A1* variations have a variety of clinical manifestations ranging from a complete female appearance to varying degrees of masculinization [13, 15]. In addition to the *NR5A1* gene, DSD can also be caused by mutations in other genes, such as the *MAP3K1* gene. As one of the common mutated genes in 46, XY DSD, *MAP3K1* variants have been showed in 13–18% of 46, XY DSD patients [16, 17]. The *MAP3K1* gene encodes a significant serine/threonine protein kinase (MAP3K1) which has a key role in cell proliferation, differentiation and apoptosis [18]. Both *NR5A1* and *MAP3K1* variants can cause 46, XY DSD, giving rise to the question of whether there is a correlation between the two molecules, which has yet to be addressed thus far.

Interestingly, we detected novel compound variants of *NR5A1* and *MAP3K1* in a Chinese patient with 46, XY DSD. Bioinformatics analysis shows that both variants are pathogenic, and in vitro experiments found the two variants can alter the expression of SOX9. Our research further confirms that the oligogenicity inheritance is an important mechanism of 46, XY DSD, and provides a reference for future research which can improve the genetic diagnosis rate of this disease.

## Methods

### Ethical approval

The Ethics Committee of Shandong Provincial Hospital affiliated to Shandong University approved this study which was conducted with the principles in the Declaration of Helsinki. And the consent obtained from all subjects included in the study and the children’s parents was both informed and written.

### Subjects

The 46, XY DSD patient with hypospadias discovered was 3 months of age in November 2018. We collected his clinical data and peripheral blood samples were drawn from the 46, XY DSD patient and this pedigree.

### Follow-up studies

We have followed this pedigree for 1 year and closely tracked following information of the patient: gonadal development, intellectual development, drug treatment, gender anxiety disorder, psychological and cognitive impairment, and the developments of gonadal tumors.

### Whole-exome sequencing and Sanger sequencing

Using the QIAamp DNA Mini Kit (Qiagen, Germany), genomic DNA was isolated from peripheral blood leukocytes following the manufacturer's instructions. WES was performed on genomic DNA. The exons were captured by using the SeqCap EZ Med Exome Enrichment Kit (Roche NimbleGen, USA) after DNA fragmentation, paired-end adaptor ligation, amplification and purification. By postcapture amplification and purification the DNA library was generated. The DNA library was sequenced on the Illumina HiSeq sequencing platform. Sequence data alignment to the human genome reference (hg19) and variant-calling were performed with NextGene V2.3.4 software to collecte the coverage and mean read depth of the target areas. The average coverage of the exome was > 100 × and the examination of the target area had enough depth to exactly match > 99% of the target exome. Variants with low coverage were filtered out to ensure the accuracy of data analysis.

Additionally, the frequency in normal populations (Genome Aggregation Database (GnomAD)) and data from the Clinvar and Online Mendelian Inheritance in Man (OMIM) databases and Human Gene Mutation Database (HGMD) were performed by using our in-house scripts and NextGene V2.3.4. We used the Standards and Guidelines for the Interpretation of Sequence Variants which was published by American College of Medical Genetics and Genomics (ACMG) and the Human Genome Variation Society (HGVS) nomenclature to determine the pathogenic variants.

Pathogenic, suspected pathogenic, or clinically ambiguous variants were detected by WES. When the suspected pathogenic or pathogenic variant was detected, we verified it by Sanger sequencing which ensured that the coverage of coding sequence can reached 100%. Tagged sequencing primers of *NR5A1* and *MAP3K1* were designed by using Primer3 version 1.1.4 (http://www.sourceforge.net) and GeneDistiller 2014 (http://www.genedistiller.org/). Forward primer (*NR5A1*): 5′-TAGTTGGGTCTCAGTGGGAGGAG-3′; Reverse primer (*NR5A1*): 5′-TCACAGAGGGTTTGGGCCAGTG-3′; Forward primer (*MAP3K1*): 5′-TCCTGAGCAGCCTTGTCTAATCT-3′; Reverse primer (*MAP3K1*): 5′-ATGTGTCATTCTTGAGGAGGGTG-3′. Polymerase chain reaction (PCR) was performed in a 50 μL system: 5 μL 10 × PCR buffer, 4 μL dNTPs, 4 μL genomic DNA, 1 μl forward and reverse primers and 0.3 μL Taq Hot Start (Takara Bio, Ohtsu, Japan). The PCR conditions contained a denaturation step (94 °C for 5 min), followed by 40 cycles of denaturation (94 °C for 30 s), annealing (65 °C for 30 s) and elongation (72 °C for 30 s). An ABI 3730 system (Applied Biosystems, Foster City, Calif., USA) was used to sequence the amplicons, and sequence analysis was finished by visual inspection and the auto assembler software Chromas 2.6.6 (Technelysium Pty Ltd. Available at www.technelysium.com.au/chromas.html).

### Bioinformatic analysis

The conservation of amino acids at mutated positions was confirmed by Clustal W (UCD, Dublin, Ireland) software. We used online software Mutation Taster (http://www.mutationtaster.org/) to predict the potential pathogenic effects of the identified variants. The model of wild type and variant proteins was constructed with I-TASSER software (https://zhanglab.ccmb.med.umich.edu/I-TASSER/).

### Site-direct mutagenesis, cloning and transient transfection

By GeneArt Gene Synthesis (Thermo Fisher Scientific, Rockford, IL), wild type *NR5A1* or *MAP3K1* cDNA were synthesized and cloned into pcDNA3.1 vector. *NR5A1* variants (pcDNA3.1-*NR5A1*-MU) and *MAP3K1* variants (pcDNA3.1-*MAP3K1*-MU) were obtained by site-directed mutagenesis. To obtain the *NR5A1* His310Pro amino acidic substitution (from adenine to cytosine at nucleotide 929), we used the following primers: primer forward ACGGGCCCTCTAGACTCGAGCGCCACCATGGACTATTCGTACGACGAG, primer reverse AGTCACTTAAGCTTGGTACCGAAGTCTGCTTGGCTTGCAGCATTTC. To obtain the *MAP3K1* Ile761Thr amino acidic substitution (from thymine to cytosine at nucleotide 2282) the corresponding primers were used: primer forward ACGGGCCCTCTAGACTCGAGCGCCACCATGGCGGCGGCGGCGGGGAATCGCGCC, primer reverse AGTCACTTAAGCTTGGTACCGACCATGTAGTACGAAAGACTGGATGCTTC. A detailed protocol used for site-directed mutagenesis can be available upon request. All the used primers were purchased from GenScript (Cayman Islands, UK). The presence and the fidelity of pcDNA3.1-*NR5A1*-MU and pcDNA3.1-*MAP3K1*-MU were confirmed by the ABI 3730 sequencing system (Applied Biosystems, Foster City, Calif., USA).

Then, the human embryonic kidney 293T/17 (HEK293T/17) cells, obtained from the National Collection of Authenticated Cell Cultures, were cultured in the high glucose Dulbecco's Modified Eagle's Medium containing 10% Fetal Bovine Serum, 5% penicillin–streptomycin and 2 mM l-glutamine. Using Lipofectamine 3000 transfection reagent (Thermo Fisher Scientific), HEK293T/17 cells were transiently transfected with plasmids containing the wild type *NR5A1* (pcDNA3.1-*NR5A1*-WT) and *MAP3K1* (pcDNA3.1-*MAP3K1*-WT) or pcDNA3.1-*NR5A1*-MU and pcDNA3.1-*MAP3K1*-MU. As previously described [19], the transfection concentration of a single plasmid was 2.5 ug/mL and the concentration of each plasmid is 1.5 ug/mL at the cotransfection of the two plasmids.

After 48 h, cells were lysed by mammalian protein extraction reagent (ThermoFisher Scientific) containing protease inhibitor cocktail (Sigma Aldrich). The HEK293T/17 cells lysates were used to analyze the protein expression of SOX9 by Western blot.

### Immunoblotting

The cells lysates mixed with Laemmli buffer containing 2-mercaptoethanol were boiled for 5 min at 99℃. Proteins were separated by sodium dodecyl sulfate polyacrylamide gel electrophoresis (SDS-PAGE; 200 V, 45 min) and then transferred onto a nitrocellulose membrane (400 mA, 1 h). Membranes were incubated overnight with primary antibodies and washed 3 times for 10 min with 0.2% tris-buffered saline Tween. Then, membranes had been incubated with secondary antibodies for 1 h and washed 3 times for 10 min with 0.2% tris-buffered saline Tween. Next, 5-min incubation between chemiluminescent HRP substrate (Millipore Corporation, Billerica, USA) and membranes were conducted and bands were visualized by Chemidoc XRS System (Bio-Rad, Hercules, CA). The following antibodies were used: rabbit anti-SOX9 (Abcam) and mouse anti-Actin (Servicebio).

## Results

### Clinical manifestation

**Fig. 1:**
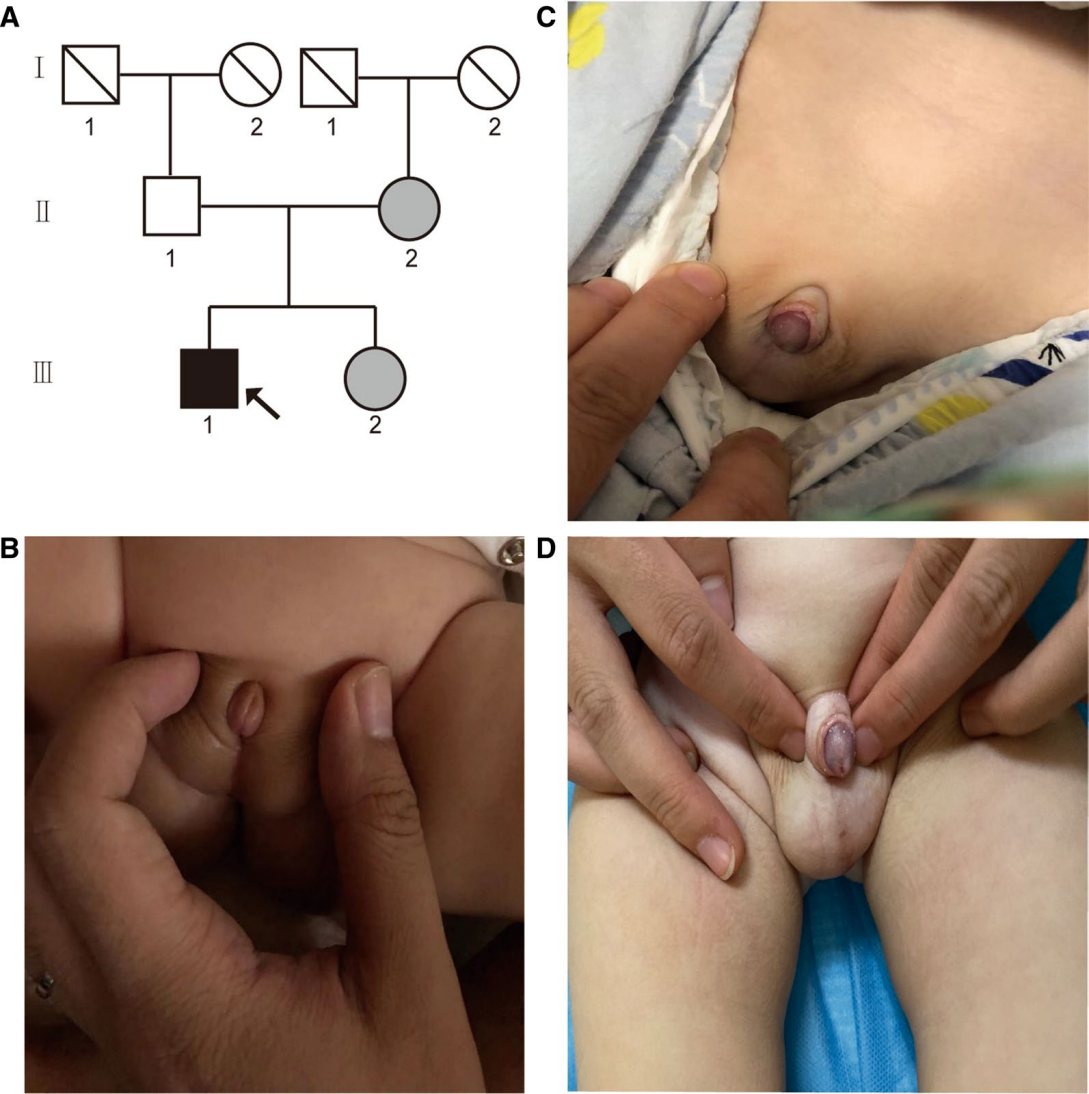
The data of the patient. **a** The pedigree including the patient. The arrow refers to the proband. Black indicates that the person has 46, XY DSD. The shading indicates that the person carries by the *MAP3K1* variant but has a healthy phenotype. The slash indicates that the person has passed away. Circles indicate females reared, and squares indicate males reared. **b** External sexual organs at 3 months of age. **c** External sexual organs at 13 months of age. **d** Picture of the patient’s external sexual organs at the last follow-up

The proband was admitted to our hospital at 3 months of age due to the perineoscrotal hypospadias. He was the second child born to a full-term pregnancy mother. The patient's lineage is shown in Fig. 1a. Family history: The proband's parents and his sister have remained phenotypically healthy to the present and denied any history of familial genetic diseases.

The genital examination showed that the penis was short, the penis head was slightly curved to the ventral side, and the foreskin was stacked on the back side of the penis head to form a headscarf, which could be turned up to reveal part of the penis head. The ventral foreskin and foreskin ligaments were absent. The penis head is flat with a shallow concave area in front. The outer urethra is located in the perineum, and the membranous proximal urethra has no urethral sponge. The central part of the scrotum is slightly divided, and the root of the scrotum extends above the root of the penis. Both testes are accessible, and their size, shape and position are normal (Fig. 1b). Testicular ultrasound (3 months old): the left testicle can be moved up to the left groin area; the right testis was located in the right scrotum; there was no echo zone at the top of the bladder.

The karyotype analysis revealed 46, XY in 50 metaphases. Hormone measurements showed a baseline serum testosterone (T) level of 0.1 ng/ml, a post-human chorionic gonadotropin (HCG) peak level of 0.46 ng/ml, 4.6 times of baseline T level; a base dihydrotestosterone (DHT) of 33.78 pg/ml, and a post-HCG peak level of 79.23 pg/ml, 2.3 times of baseline DHT level. This indicated that testosterone can respond to stimuli but the basal value of hormones was low, and it was necessary to continue follow-up to observe changes in hormone levels of our patient. Serum AMH was 17.7 ng/ml (Table 1). The luteinizing hormone releasing hormone (LHRH) stimulation test at 9 months showed that the patient's pituitary response was normal (Additional file 1).

**Table. 1:** Hormone measurements of the patient

Months	LH (mIU/L)	FSH (mIU/L)	T (ng/ml)	E2 (pg/ml)	DHT (pg/ml)	AMH (ng/ml)	T resp. hCG (ng/ml)	DHT resp. hCG (pg/ml)	PRL (ng/ml)	PRG (mg/ml)
3	0.5	4.54	0.1	20	20.66	17.7	–	–	8.93	1.26
4	–	–	0.1	–	33.78	–	0.46	79.23	–	–
9	2.31	12.15	0.93	20	–	–	–	–	13.13	–

*AMH* anti Müllerian hormone, *DHT* dihydrotestosterone, *E2* estradiol, *FSH* follicle stimulating hormone, *hCG* human chorionic gonadotropin, *LH* luteinizing hormone, *PRL* prolactin, *PRG* progesterone, *T* testosterone

### Follow up

At 11 months of age, the proband underwent hypospadias repair. After the operation, the patient was referred to our pediatric endocrine clinic at 13 months of age (Fig. 1c).

The proband's last follow-up was at 1.3 years of age and the proband identified as male. Gynecological examination confirmed male external sexual organs (Fig. 1d). Testicular ultrasound (1.3 years old): The echo of testicles could be detected in both scrotums. The morphology of testicles on both sides was normal. The size of testicles on the left side was 1.7 cm × 0.8 cm × 0.7 cm, and that on the right side was 1.5 cm × 0.9 cm × 0.6 cm. A fluid zone is seen around the right testicle, about 0.6 cm deep. During the follow-up period, the proband's mental development was normal and no medication was used. In addition, potential complications, psychological or cognitive impairment, gonadal tumors or gender anxiety disorder were not observed.

### Mutation detection

**Fig. 2:**
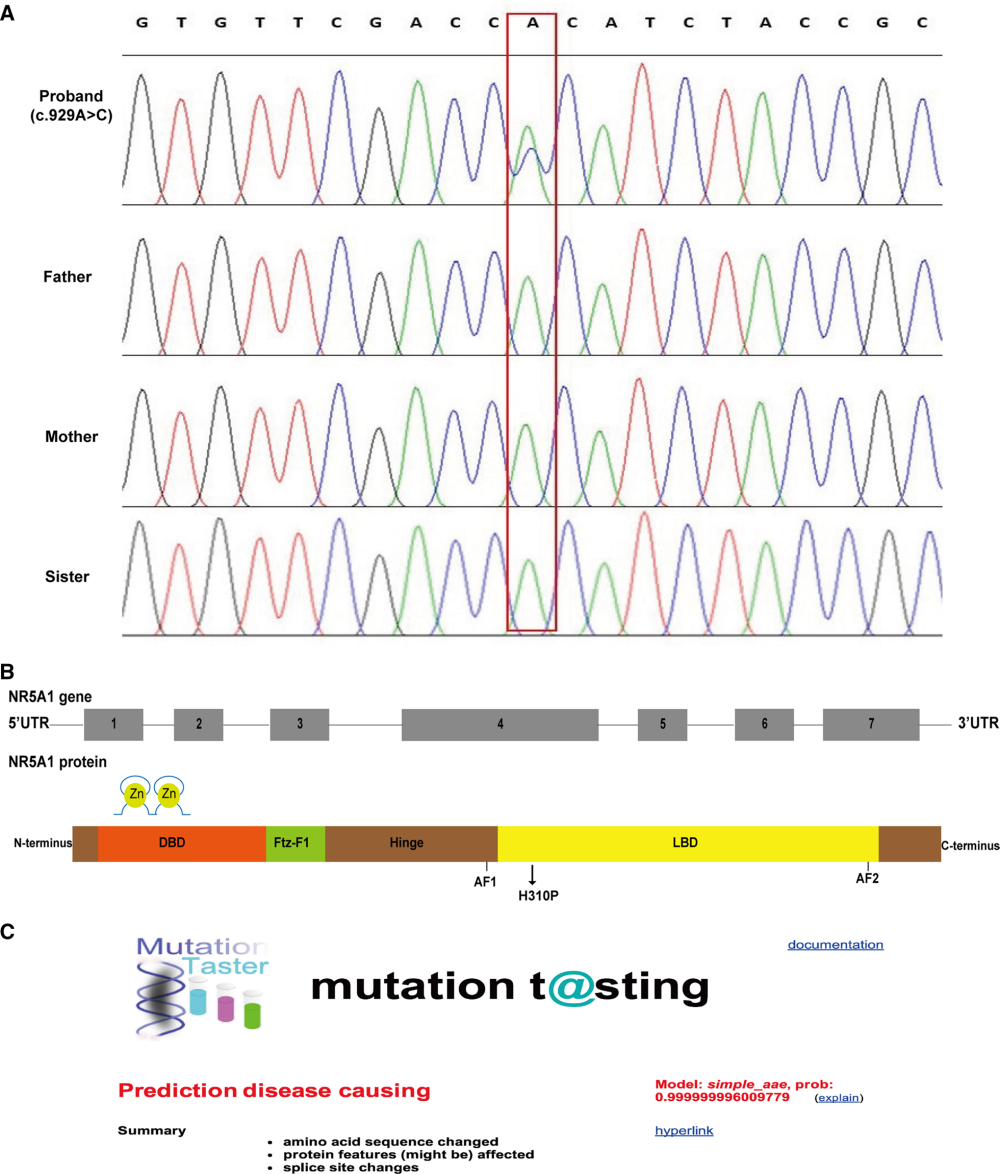

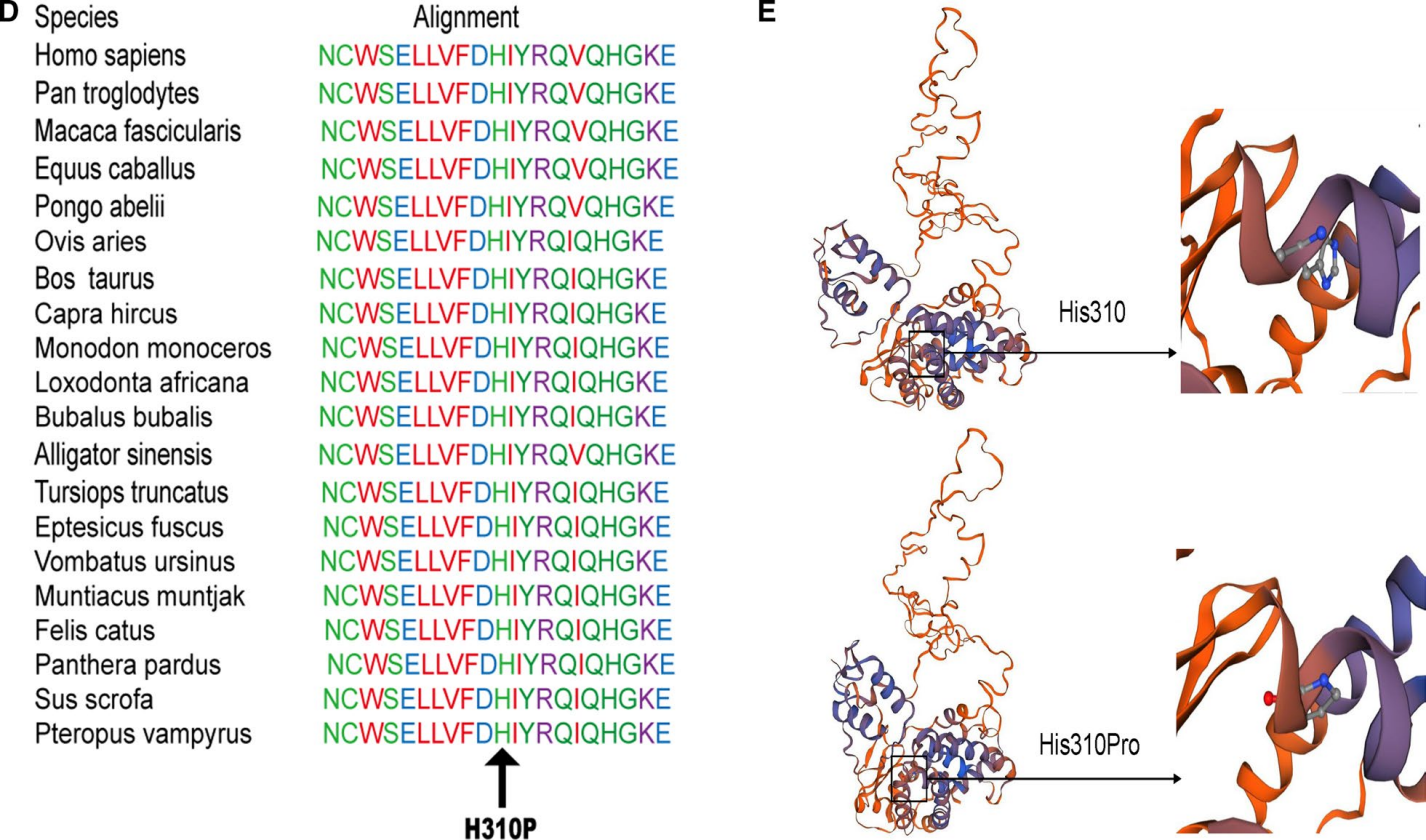
Sequencing results and bioinformatic analysis of the *NR5A1* gene variant identified in our study. **a** Partial sequence diagram of *NR5A1* in our case. A novel heterozygous variant in *NR5A1* gene (c.929A > C) of II-1, resulting in the 310th amino acid of the encoded protein from histidine to proline, is shown by an arrow. **b** Location and structure NR5A1. The *NR5A1* gene consists of one non-coding exon (exon 1) and six coding exons (exons 2–7). It encodes SF-1, consisting of 461 amino acids, which contains a DNA binding domain (DBD), a ligand binding domain (LBD), two function activation domains (AF-1 and AF-2), an auxiliary region and a hinge region. This novel variant (His310Pro) is located in the LBD and is predicted to cause a ligand-NR5A1-binding disorder and lead to NR5A1 dysfunction in sex differentiation, sexual development and steroidogenesis. **c** Prediction of the novel damaging variant c.929A > C (p. His310Pro) in MutationTaster. **d** Cross-species conservation of NR5A1 around p. His310 is displayed. **e** Protein structure prediction of wild-type and mutant NR5A1

We subsequently applied the WES to the genetic analysis of the pedigree and found a novel heterozygous *NR5A1* variant (c.929A > C) of II-1 resulting in a change in-the 310th amino acid of the encoded protein from histidine to proline (His310Pro, Fig. 2a). No substitution was detected in GnomAD, indicating that this variant is not polymorphic. As shown in Fig. 2b, the *NR5A1* gene has one non-coding exon (exon 1) and six coding exons (exons 2–7). It encodes SF-1, consisting of 461 amino acids, which contains a DNA binding domain (DBD), a ligand binding domain (LBD), two function activation domains (AF-1 and AF-2), an auxiliary region and a hinge region. This novel variant (His310Pro) is located in the LBD and is predicted to cause a ligand-NR5A1-binding disorder and lead to NR5A1 dysfunction in sex differentiation, sexual development and steroidogenesis. MutationTaster showed that the protein properties influenced by the novel variant were pathogenic (Fig. 2c). After application of the ACMG criteria, the *NR5A1* variant is “Likely Pathogenic”. Multiple amino acid sequence alignments conducted by the Clustal W tool suggested that p. His310 is conserved across various species (Fig. 2d). This *NR5A1* variant obviously caused changes in the 3D structure of the NR5A1 protein compared to wild type (Fig. 2e).

**Fig. 3:**
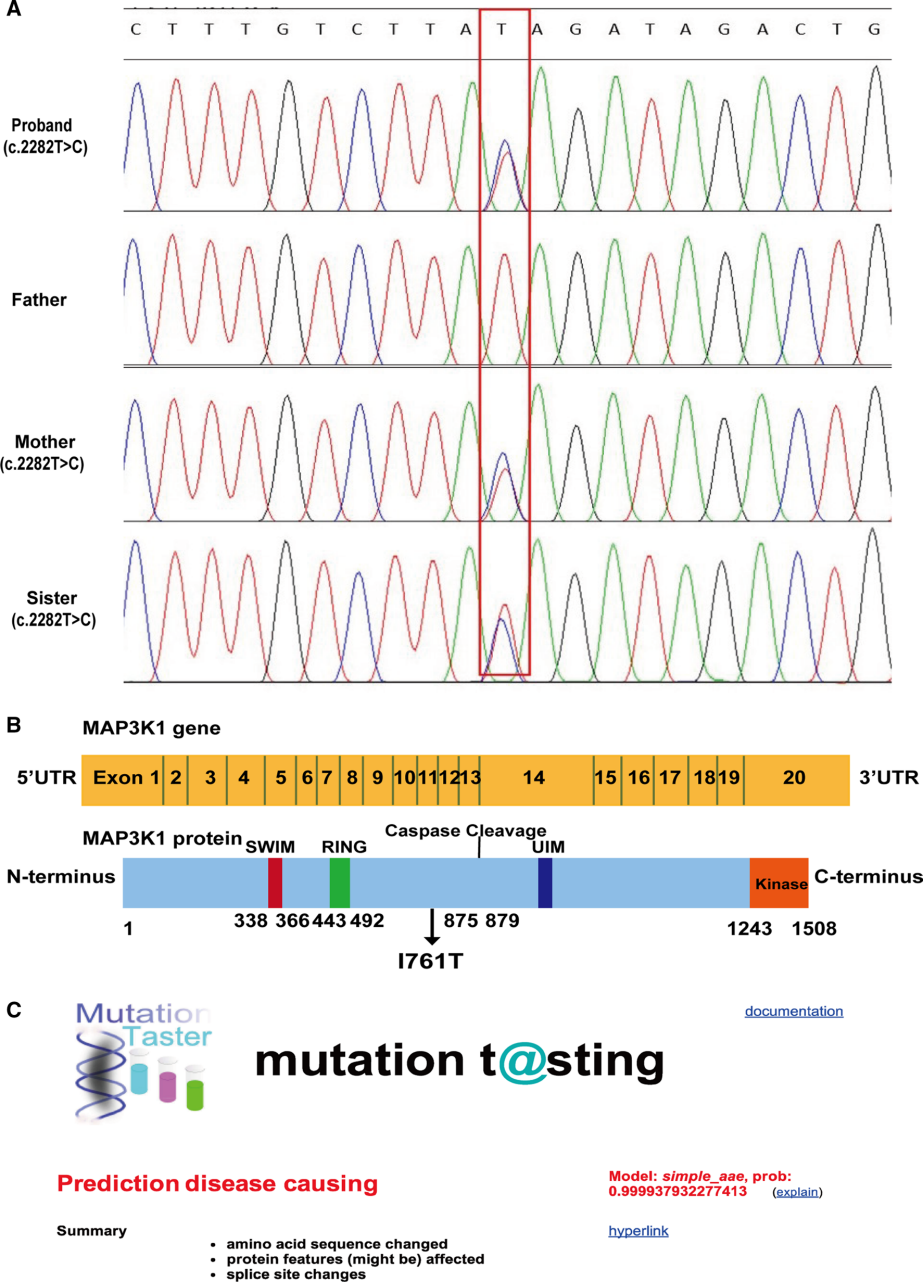

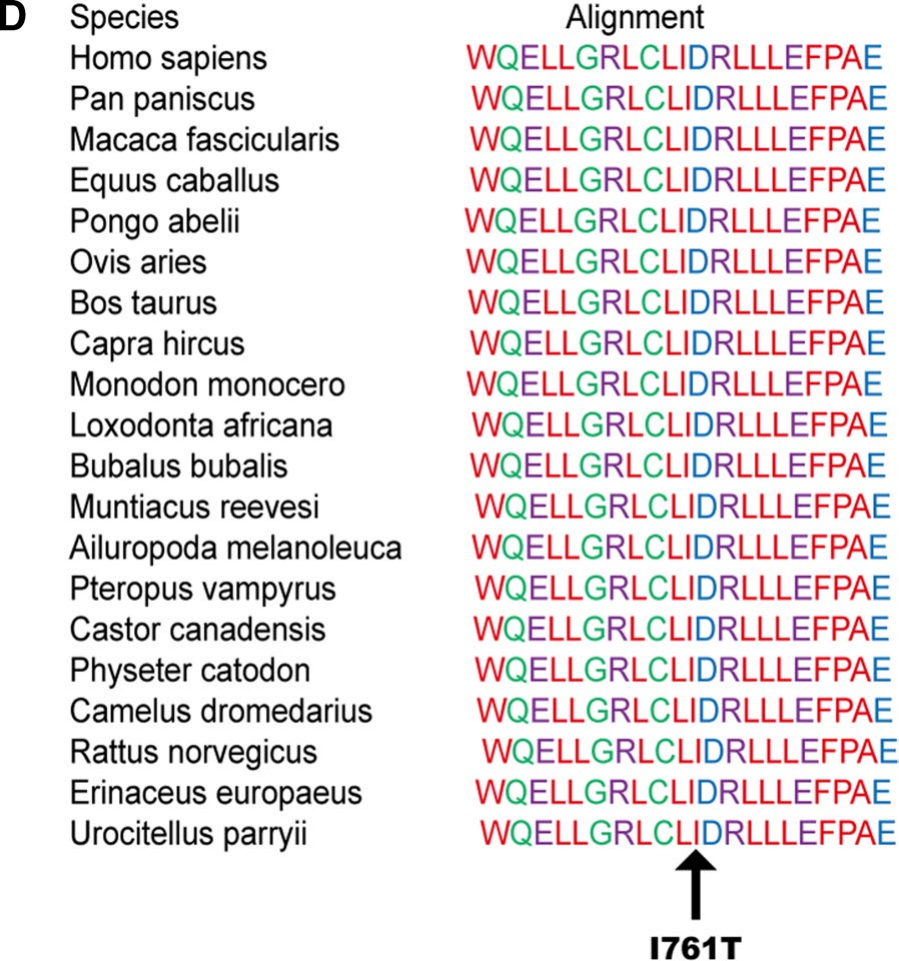
Sequencing results and bioinformatic analysis of the *MAP3K1* gene variant identified in our study. **a** Partial sequence diagram of *MAP3K1* in our case. A rare heterozygous variant in *MAP3K1* (c.2282T > C) of patient I-2, II-1 and II-2, resulting in the 761th amino acid of the encoded protein from isoleucine to threonine, is shown by an arrow. **b** The structure domains of MAP3K1. Variants at the protein level are indicated below the domains. **c** Prediction of the rare damaging variant c.2282T > C (p. Ile761Thr) in MutationTaster. **d** Cross-species conservation of MAP3K1 around p. Ile761 is displayed

A rare heterozygous *MAP3K1* variant (c.2282 T > C), which can result in a change in the 761th amino acid of the encoded protein from isoleucine to threonine (Ile761Thr, Fig. 3a), was also identified in the proband, the proband's mother and sister. The frequency of this variant in the normal person database is T = 0.00007843 (22/280512, GnomAD). The C-terminus of MAP3K1 has a conserved caspase-3 cleavage site, a ubiquitin interaction motif (UIM) and the serine/threonine kinase domain. Two zinc finger structures, one RING structure and one SWIM region, are located at the N-terminus of MAP3K1. The heterozygous variant occurs at position 761 (Ile761Thr) (Fig. 3b). MutationTaster showed that the protein properties influenced by the gene variant were pathogenic (Fig. 3c). Multiple amino acid sequence alignments conducted by the Clustal W tool suggested that p. Ile761 is conserved across various species (Fig. 3d). However, the structural analysis could not be performed due to the 3D protein of MAP3K1 cannot be crystalized currently.

### The SOX9 protein expression in HEK293T/17 cells

**Fig. 4:**
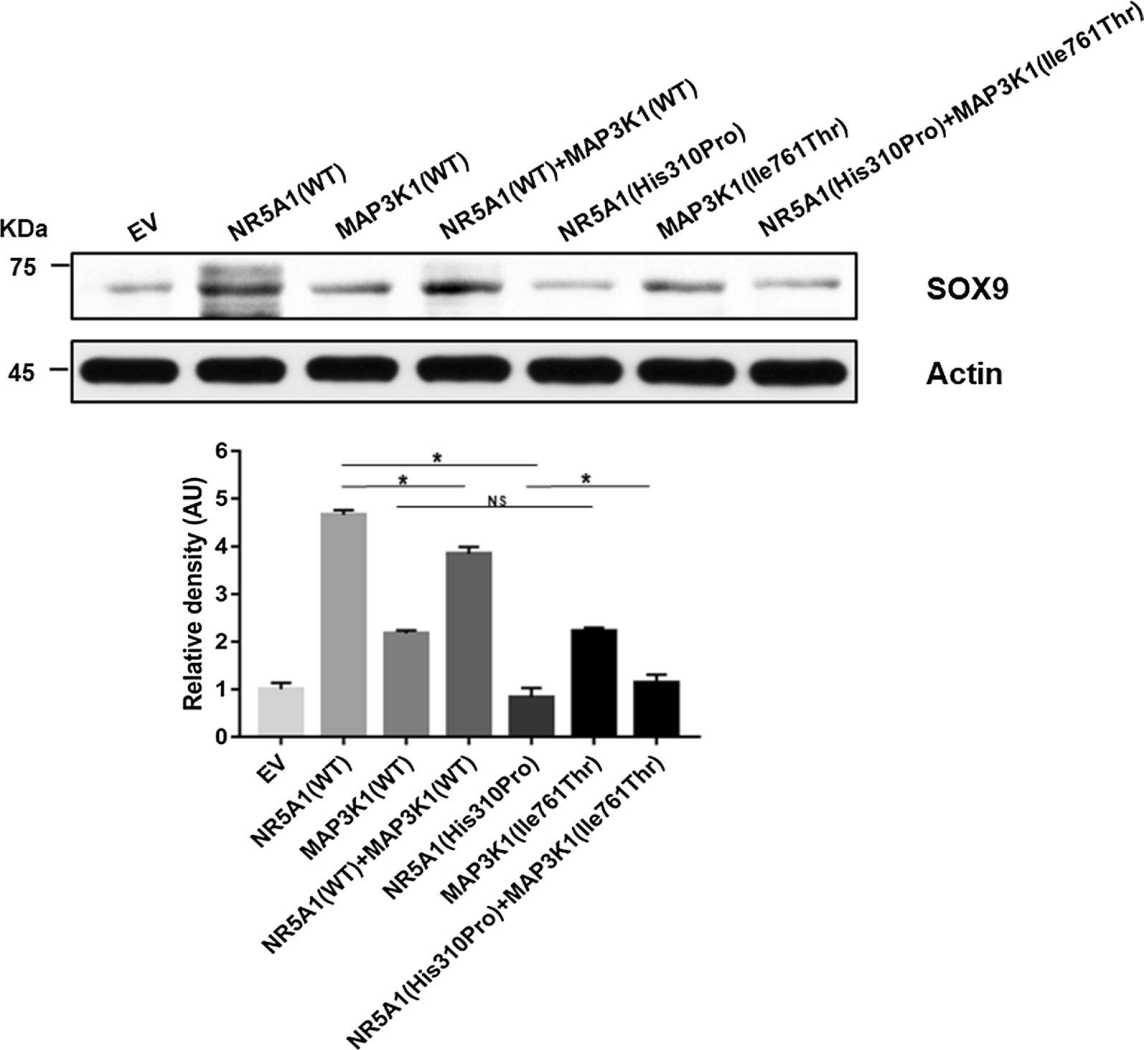
Western blotting analysis of SOX9 production in HEK 293 T/17 cells. The SOX9 production of cells transfected with the pcDNA3.1-*NR5A1*-MU decreased significantly by about 82.11% compared with that of cells transfected with pcDNA3.1-*NR5A1*-WT. The single pcDNA3.1-*MAP3K1*-MU transfection had little effect on the SOX9 production compared to pcDNA3.1-*MAP3K1*-WT. Compared to the single pcDNA3.1-*NR5A1*-WT transfection, the SOX9 protein production of cells transfected with pcDNA3.1-*NR5A1*-WT and pcDNA3.1-*MAP3K1*-WT decreased about 17.40%, which indicated that *MAP3K1* can inhibit the expression of SOX9 and was consistent with our prediction. Compared to cells transfected with the pcDNA3.1-*NR5A1*-MU, the SOX9 protein production of cells transfected with both mutant plasmids increased 36.64%, which indicated that the effect of increasing SOX9 production became strong after *MAP3K1* was mutated. *EV* empty vector, *WT* wild type, *NR5A1* nuclear receptor subfamily 5 group A member 1, *MAP3K1* mitogen-activated protein kinase kinase kinase 1. *P < 0.05, *NS* no statistical significance

SOX9 plays an important role in testicular differentiation. We speculated that it is a common downstream signaling molecule of *NR5A1* and *MAP3K1*. Therefore, we detected the production of SOX9 in HEK293T/17 cells subsequently. Plasmids containing the wild type *NR5A1* and *MAP3K1* cDNA or carrying *NR5A1* and *MAP3K1* variants were transiently transfected into HEK293T/17 cells to determine the effect of the *NR5A1* and *MAP3K1* variants on SOX9. The SOX9 production of cells transfected with the pcDNA3.1-*NR5A1*-MU decreased significantly by about 82.11% compared with that of cells transfected with pcDNA3.1-*NR5A1*-WT. The single pcDNA3.1- *MAP3K1*-MU transfection had little effect on the SOX9 production compared to pcDNA3.1- *MAP3K1*-WT. Compared to the single pcDNA3.1-*NR5A1*-WT transfection, the SOX9 protein production of cells transfected with both pcDNA3.1-*NR5A1*-WT and pcDNA3.1- *MAP3K1*-WT decreased by about 17.40%, which indicated that *MAP3K1* can inhibit the expression of SOX9 and was consistent with our prediction. Compared to cells transfected with the pcDNA3.1-*NR5A1*-MU, the SOX9 protein production of cells transfected with both variant plasmids increased by the 36.64%, which indicated that the effect of stimulating SOX9 production became strong after *MAP3K1* was mutated (Fig. 4).

## Discussion

In the present study, we described a 46, XY DSD patient with a novel heterozygous *NR5A1* variant (c.929A > C, p. His310Pro) and a rare heterozygous *MAP3K1* variant (c.2282T > C, p. Ile761Thr). These two variants were predicted to be pathogenic by bioinformatics analysis. At the protein level, we characterized the pathogenic *NR5A1* and *MAP3K1* variants can affect the production of SOX9. Therefore, we hypothesize that these two identified variants are the exclusive etiology of the phenotype in our patient. Moreover, digenic inheritance may play a vital role in the phenotype spectrum of 46, XY DSD associated with *NR5A1* variants.

The *NR5A1* gene (OMIM 184757), located at 9q33, extends more than 30 kb of genomic DNA and plays a central role in human sex differentiation, sexual development, and steroid production [20–22]. 46, XY DSD with *NR5A1* variants have a variety of clinical manifestations, from coplete female appearance to varying degrees of virilization. Most children present mainly with labial masses and clitoral hypertrophy that are easily misdiagnosed as AIS. Prepubertal or adolescent patients show clinical manifestations with masculinity such as micropenis, cryptorchidism, and hypospadias, which are difficult to distinguish from 5α-R2D [23]. In this study, our patient is a 13 months’ old child with short penis and hypospadias, which are different from most children with *NR5A1* variants as a labial mass and clitoral hypertrophy. We described this patient has a novel heterozygous *NR5A1* variant (c.929A > C, p. His310Pro) and a rare heterozygous *MAP3K1* variant (c.2282T > C, p. Ile761Thr) and suspected that the rare *MAP3K1* variant may accelerate the development of hypospadias.

The same variant in the *NR5A1* gene may cause significantly different degrees of virilization in different individuals, which means that it is difficult to establish a link between genotype and phenotype. One of the possible reasons that can explain this phenomenon is the oligomeric inheritance of *NR5A1* cases. Camats [15] and Mazen [24] et al. believe that there may be genetic modifiers that affect the severity of the phenotype. The p. Arg92Trp variant is described as associated with a different phenotype. For example, *SRY* negative 46, XX ovotesticular DSD and 46, XX testicular DSD [25] and *SRY* positive 46, XY partial gonadal dysgenesis [26].

As a part of the gene network, *MAP3K1* is responsible for gonadal development and involved in the regulation of cell proliferation, differentiation and apoptosis. *MAP3K1* variants can altered the binding of cofactors and increase the phosphorylation of the downstream MAP kinase pathway targets, then, the phosphorylation of ERR1/2 and p38 can be promoted and thereby 46, XY DSD occurs [27–29]. The findings of Maria et al. [30] showed that the expression of constitutively active MAP3K1 leads to the apoptosis of AR-positive cancer cells, and the reconstruction of the AR pathway makes prostate cancer cells sensitive to MAP3K1-induced apoptosis. MAP3K1 can activate the transcription factor AP-1 [31, 32], which may play a key role in the cistrome of *AR* [33], leading to the AIS heterogeneity.

To date, only three possible cases that digenic inheritance exists in 46, XY DSD associated with *NR5A1* variants have been reported. A recent study also identified pathogenic variations of *NR5A1* and *MAP3K1* in an individual with 46, XY DSD [24]. The patient carried a missense variant previously reported in *NR5A1* (c.937C > T, p. Arg313Cys) and a rare *MAP3K1* variant (c.710A > G, p. Gln237Arg) (Table. 2). The *NR5A1* variant not carried by either parent is new, while the *MAP3K1* variant was inherited from the mother. This patient was a 4-year-old female with ambiguous external genitalia. Genital examination found that distinct gonads were in the inguinal region and pelvic sonar confirmed the presence of both testicles in the inguinal region. A urethral opening was at the tip of the swollen clitoris. Karyotype analysis revealed 46, XY in 50 metaphases. The HCG test showed a poor testosterone response and LHRH stimulation tests revealed that the pituitary can respond to stimulation. The authors suggested that the rare *MAP3K1* variants leading to abnormal development of testes may alter the activity of MAP kinase and/or cofactor binding above threshold.

**Table 2:** Reported patients with digenic inheritance of 46, XY DSD associated with *NR5A1* variants

Gene	*NR5A1*/*ZFPM2*	*NR5A1/SRD5A2*	*NR5A1*/*MAP3K1*	*NR5A1*/*MAP3K1*
Coding change	c.G251A/c. A2107C	c.1114_1116del^/c. G680A	c.937C > T/c.710A > G	c.929A > C/c.2282T > C
Protein change	p. Arg84His/p. Met703Leu	p. Lys372del^/p. Arg227Gln	p. Arg313Cys/p. Gln237Arg	p. His310Pro/p. Ile761Thr
Zygosity	Heterozygous/Heterozygous	Heterozygous/Heterozygous	Heterozygous/Heterozygous	Heterozygous/Heterozygous
External genitalia	Clitoromegaly one perineal opening	Proximal hypospadias	Swollen clitoris, urethral openings at tip	hypospadias
Gonadal location	Bilateral inguinal region	Bilateral scrotum	Bilateral inguinal region	Scrotum
Müllerian structures	Not	Not	Not	Not
Basal testosterone	T&DHT markedly decreased	T-elevated, DHT-normal	T&DHT markedly decreased	T&DHT markedly decreased
Basal gonadotrophins	LH-elevated, FSH-elevated	LH-normal, FSH-elevated	LH-decreased, FSH- decreased	LH-decreased, FSH-normal
T resp. hCG	No response	Elevated	Poor response	Elevated
LH resp. hCG	ND	ND	Elevated	Elevated
FSH resp. hCG	ND	ND	Elevated	Elevated
AMH	Decreased	Decreased	Elevated	Elevated
Adrenal function	Normal	Normal	Normal	Normal
Reference	Gorjana, et al., 2017	Gorjana, et al., 2017	Inas, et al., 2016	Novel

*AMH* anti Müllerian hormone, *T* testosterone, *DHT* dihydrotestosterone, *FSH* follicle stimulating hormone, *LH* luteinizing hormone, *ND* not done, *hCG* human chorionic gonadotropin

Gorjana et al. also identified two patients with two variants of testis-determining genes, the pathogenic variations of *NR5A1* and *ZFPM2*, and the pathogenic variations of *NR5A1* and *SRD5A2*, respectively [34] (Table 2). Firstly, the study identified two patients with same *NR5A1* variation (c. G251A, p. Arg84His), but they had significantly different clinical phenotypes. The testes of patient only with the variation of *NR5A1* in the inguinal gonads had some function, while patient with the variations of *NR5A1* (c. G251A, p. Arg84His) and *ZFPM2* (c. A2107C, p. Met703Leu) had more severe clinical manifestations: no testosterone production, orchiatrophy, and in situ germ cell tumor formation. The researchers hypothesized that the accumulative effect of two different variants might be the cause of the more severe clinical phenotype of patient with double gene variants. Secondly, another patient who had *NR5A1* variants (c.1114_1116del^, p. Lys372del^) and *SRD5A2* variants (c. G680A, p. Arg227Gln) presented with proximal hypospadias and bilateral scrotal gonads. The findings further highlighted the possibility that digenic inheritance may play a significant role in the phenotype heterogeneity associated with the *NR5A1* variant in 46, XY DSD. ZFPM2 protein is a polytype zinc finger cofactor, which can bind to the N-terminal zinc finger of Gata4 protein, and can act as a transcriptional co-activator or co-inhibitor according to cell environment and target genes [35, 36]. Gata4 is an evolutionarily conserved transcription factor and is essential for the early development of multiple organs including the testis. A direct interaction between Gata4 and ZFPM2 proteins may be necessary for appropriate levels of *SRY* expression. Anu B, et al. [37] demonstrated for the first time in humans, as in mice, that testicular dysplasia was associated with *ZFPM2* variants that alter its ability to regulate expression of target genes in gonad development. Yuka et al. [38] found that the homologous proteins Six1 and Six4 play a crucial role in gonad development by regulating downstream targets *ZFPM2* and *NR5A1*. The Six1 and Six4 genes belong to mammalian homologs of the Drosophila sine homology box (Six) family. Six1 and Six4 are located in the same genomic region and have highly overlapping tissue expression during mouse embryogenesis [39]. Six1/Six4 transactivates *NR5A1*, which is connected with the initial growth of gonadal precursor cells before *SRY* expression begins. Second, they activate *ZFPM2*, which is related to the upregulation of *SRY* in male gonad differentiation.

**Fig. 5:**
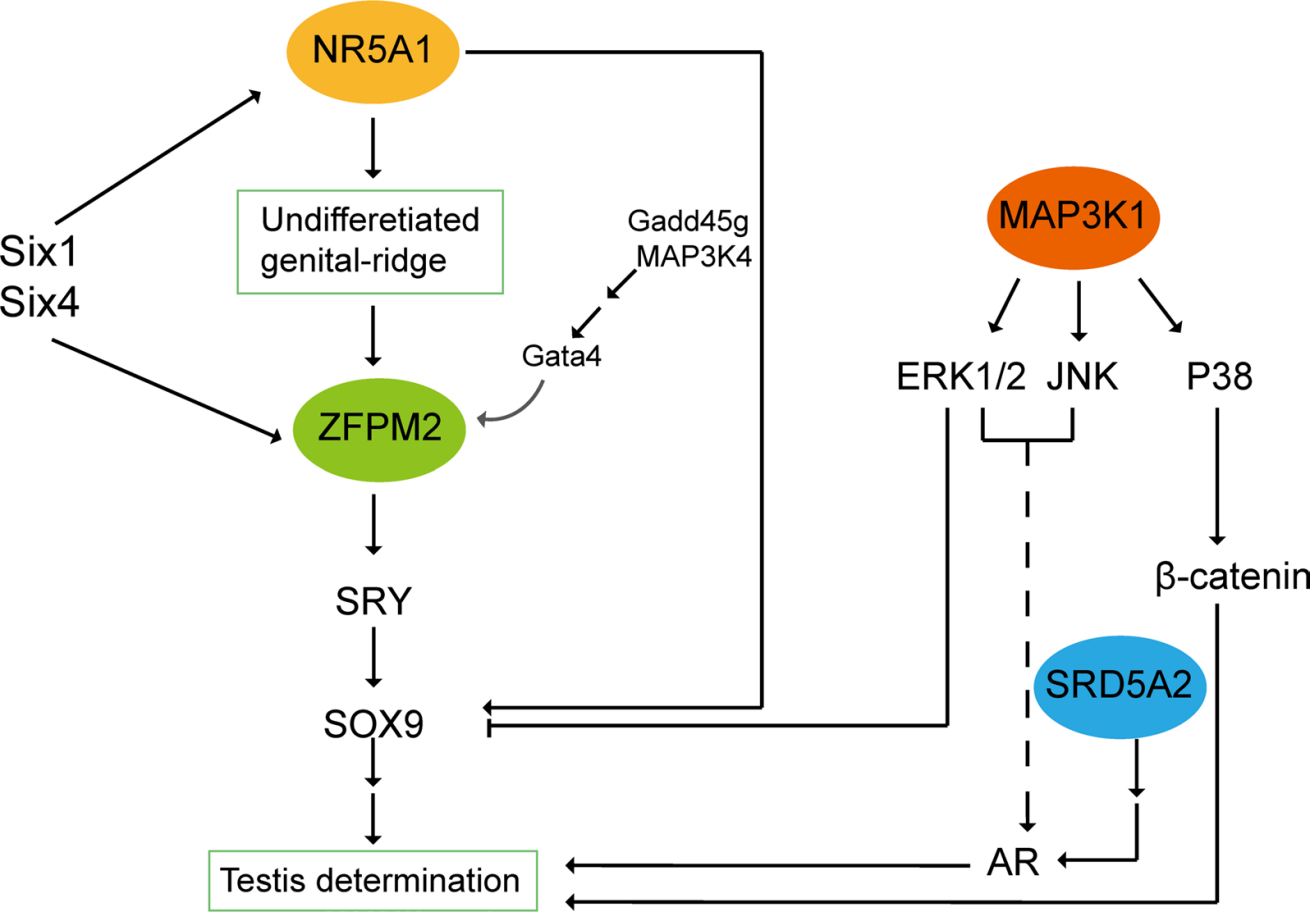
Prediction of the relationship between *NR5A1* and *MAP3K1*, *ZFPM2*, *SRD5A2*. *SOX9* Sry-box 9, *ERK* extracellular signal-regulated kinase, *JNK* c-Jun NH2-terminal kinase, *AR* androgen receptor, *SRD5A2* steroid 5 reductase 2

The relationship between *NR5A1* and *MAP3K1*, *ZFPM2*, *SRD5A2* are shown in Fig. 5. Studies have shown that the homologous proteins Six1 and Six4 can regulate male sex determination and mouse gonad development through *NR5A1* (*AD4BP/SF1*) and *ZFPM2* (*Fog2*). In the regulation of male sex determination, *ZFPM2* induces *SRY* expression, while *NR5A1* determines the growth of initial gonadal precursor cells in the regulation of gonadal precursor formation. *ZFPM2* interacts with activated *Gata4*, which may be activated by phosphorylation through the Gadd45g/MAP3K4 pathway. Activated *Gata4* interacts with *ZFPM2* to activate *SRY* and induce gonadal differentiation into testes [38, 40]. *MAP3K1* variants can altered binding of cofactors and increase the phosphorylation of the downstream MAP kinase pathway targets-MAPK11, MAP3K and MAPK1, thereby promoting the phosphorylation of ERR1/2 and p38 and ultimately leading to the occurrence of 46, XY DSD. Increased phosphorylation of ERK1/2 and p38 may result in decreased expression of SOX9 and increased activity of β-catenin, respectively, which are important signaling molecules in testicular and ovarian-promoting pathways [27–29]. *NR5A1* and *MAP3K1* can promote or inhibit the expression of SOX9 through downstream signal transduction pathways, respectively, and ultimately lead to male sex determination and differentiation.

In addition, *MAP3K1* is expected to regulate the expression of *AR* gene [19, 31, 33], and whether *MAP3K1* and *SRD5A2* (steroid 5 reductase 2, OMIM 607306) [41, 42] interact through *AR* is worthy of further study.

To explain the underlying molecular mechanism of our genetic results, we conducted in vitro experiments of the *NR5A1* and *MAP3K1* variants and the accumulative effect of two different variants might be the cause of the clinical phenotype of our patient. Moreover, the pattern of inheritance of previously reported pathogenic *MAP3K1* variants is autosomal dominant and sex-limited [16, 43]. *MAP3K1* variants were first described in 2010 in two large families and two of 11 sporadic cases with 46, XY DSD and an autosomal dominant, sex-limited pattern of transmission [16]. In the large family reported, phenotypic manifestations range from complete hypogonadism to hypospadias and cryptorchidism. However, 46, XX carriers were unaffected. Granados A, et al. [43] reported six individuals from four unrelated families that had pathogenic *MAP3K1* variants causing both partial and complete gonadal dysgenesis. Similarly, 46, XX females carrying *MAP3K1* variants are unaffected. 46, XY DSD has a sex-limited autosomal dominant mode of inheritance affecting only XY individuals [44]. As the above studies, the mothers and sister of 46, XX in our study have the *MAP3K1* variant but remain phenotypically healthy to the present. Based on the discussion above, we have reasons to consider that this variant is pathogenic and pathogenic *MAP3K1* variants has the pattern of inheritance of sex-limited autosomal dominant.

Notably, serum AMH level can reflect the health and number of Sertoli cells (SCs), the maturity of SCs and the exposure to FSH and intra-tubular testosterone [45]. The levels of serum AMH depend on the number and completeness of SCs [46]. Therefore, the reduced serum AMH level in our patient may have a special suggestive effect in the case of SCs dysfunction [47].

It is undeniable that our study has some limitations. The grandparents of our 46, XY Chinese patient had died and we cannot trace potential origin of the genetic variation. In addition, the basic experiments to validate interaction mechanism between the *NR5A1* and *MAP3K1* gene are temporarily lacking. Our results extend the *NR5A1* and *MAP3K1* variant profile. However, a lot is still unknown about the association between the phenotype and genotype of 46, XY DSD, and more 46, XY DSD patients need to be recruited for future research.

## Conclusions

In summary, our study identified novel compound variants of the *NR5A1* and *MAP3K1* genes which can alter the expression of SOX9 and ultimately resulted in a specific phenotype of a patient with 46, XY DSD. Digenic inheritance may play a key role in the phenotypic spectrum of 46, XY DSD associated with *NR5A1* variants.

## Supplementary Information

**Additional file 1** The luteinizing hormone releasing hormone (LHRH) stimulation test at 9 months. The results showed that the patient's pituitary response was normal

## Data Availability

All the data can be available from the corresponding author upon reasonable request.

## Abbreviations

46, XY DSD: 46, XY differences in sex development;
AIS: androgen insensitivity syndrome
SF-1: steroidogenic factor 1
SOX9: SRY-box transcription factor 9
AMH: anti-Müllerian hormone
ERK: extracellular signal-regulated kinase
JNK: C-Jun NH2-terminal kinase
WES: whole exome sequencing
GnomAD: genome genome aggregation database
HGMD: human gene mutation database
OMIM: online Mendelian inheritance in man
ACMG: American College of Medical Genetics and Genomics
HGVS: Human Genome Variation Society
PCR: polymerase chain reaction
T: testosterone
HCG: human chorionic gonadotropin
DHT: dihydrotestosterone
LHRH: luteinizing hormone releasing hormone
DBD: DNA binding domain
LBD: ligand binding domain
NTD: N-terminal domain
UIM: ubiquitin interaction motif
5α-R2D: 5α-Reductase type 2 deficiency
AR: androgen receptor
SRD5A2: steroid 5 reductase 2
