# Supplementary figures and images for: Characteristics and possible mechanisms of 46, XY differences in sex development caused by novel compound variants in NR5A1 and MAP3K1
Source: Orphanet J Rare Dis. 2021 Jun 10;16:268. doi: 10.1186/s13023-021-01908-z (PMC8194036; doi:10.1186/s13023-021-01908-z)

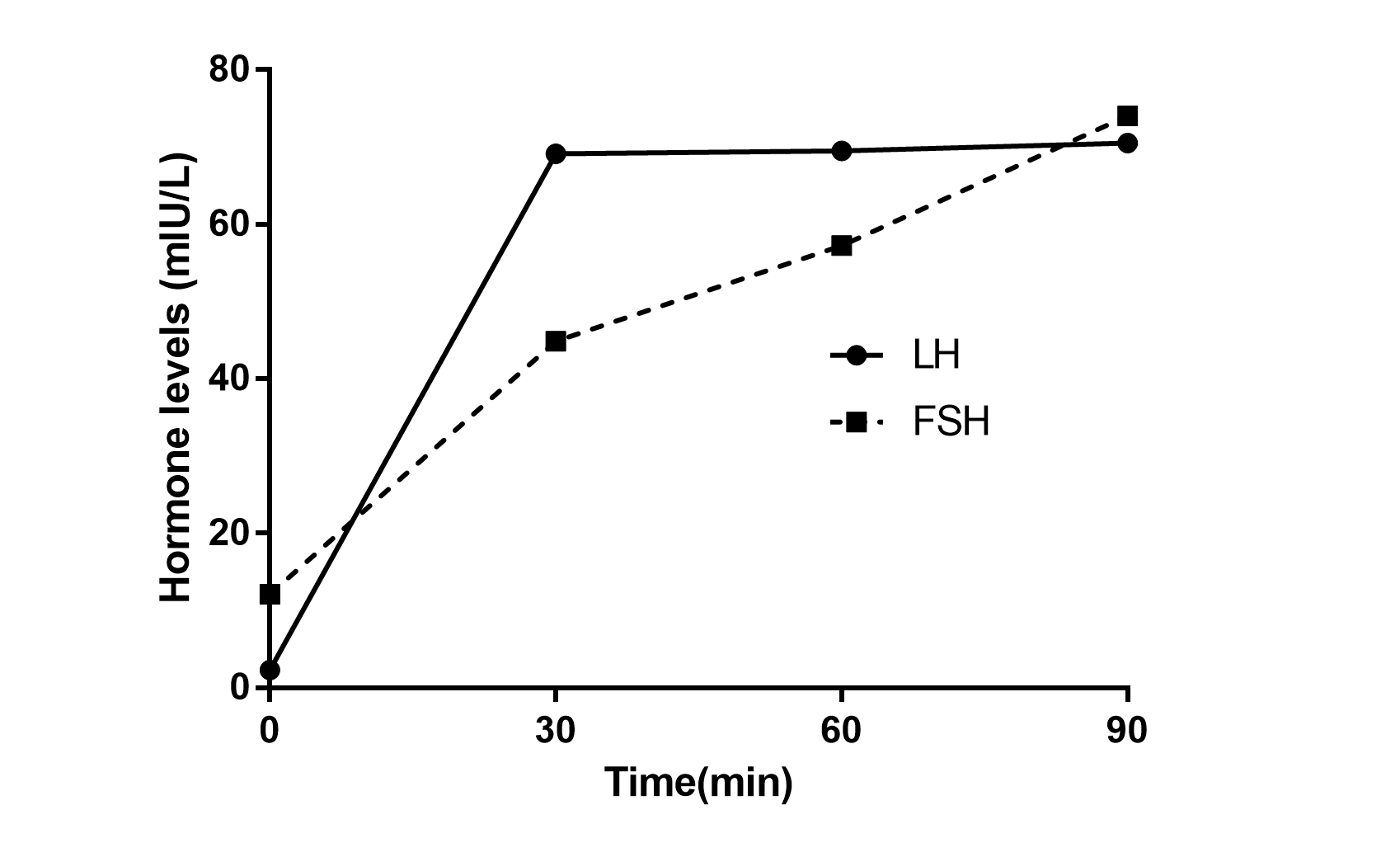

Supplement: Supplementary file 1 — Additional file 1 The luteinizing hormone releasing hormone (LHRH) stimulation test at 9 months. The results showed that the patient's pituitary response was normal [file 13023_2021_1908_MOESM1_ESM.tif]
